# Facilitating implementation of organs-on-chips by open platform technology

**DOI:** 10.1063/5.0063428

**Published:** 2021-10-12

**Authors:** Anke R. Vollertsen, Aisen Vivas, Berend van Meer, Albert van den Berg, Mathieu Odijk, Andries D. van der Meer

**Affiliations:** 1 Applied Stem Cell Technologies, University of Twente, Enschede, the Netherlands; 2 BIOS Lab on a Chip Group, MESA+ Institute for Nanotechnology, Max Planck Institute for Complex Fluid Dynamics, University of Twente, Enschede, the Netherlands; 3 Organ-on-Chip Center Twente, University of Twente, Enschede, the Netherlands; 4 Department of Anatomy and Embryology, Leiden University Medical Centre, Leiden, the Netherlands

## Abstract

Organ-on-chip (OoC) and multi-organs-on-chip (MOoC) systems have the potential to play an important role in drug discovery, disease modeling, and personalized medicine. However, most devices developed in academic labs remain at a proof-of-concept level and do not yet offer the ease-of-use, manufacturability, and throughput that are needed for widespread application. Commercially available OoC are easier to use but often lack the level of complexity of the latest devices in academia. Furthermore, researchers who want to combine different chips into MOoC systems are limited to one supplier, since commercial systems are not compatible with each other. Given these limitations, the implementation of standards in the design and operation of OoCs would strongly facilitate their acceptance by users. Importantly, the implementation of such standards must be carried out by many participants from both industry and academia to ensure a widespread acceptance and adoption. This means that standards must also leave room for proprietary technology development next to promoting interchangeability. An open platform with standardized interfacing and user-friendly operation can fulfill these requirements. In this Perspective article, the concept of an open platform for OoCs is defined from a technical perspective. Moreover, we discuss the importance of involving different stakeholders in the development, manufacturing, and application of such an open platform.

## I. INTRODUCTION: ORGAN-ON-CHIP DEVELOPMENT

**FIG. 1.**
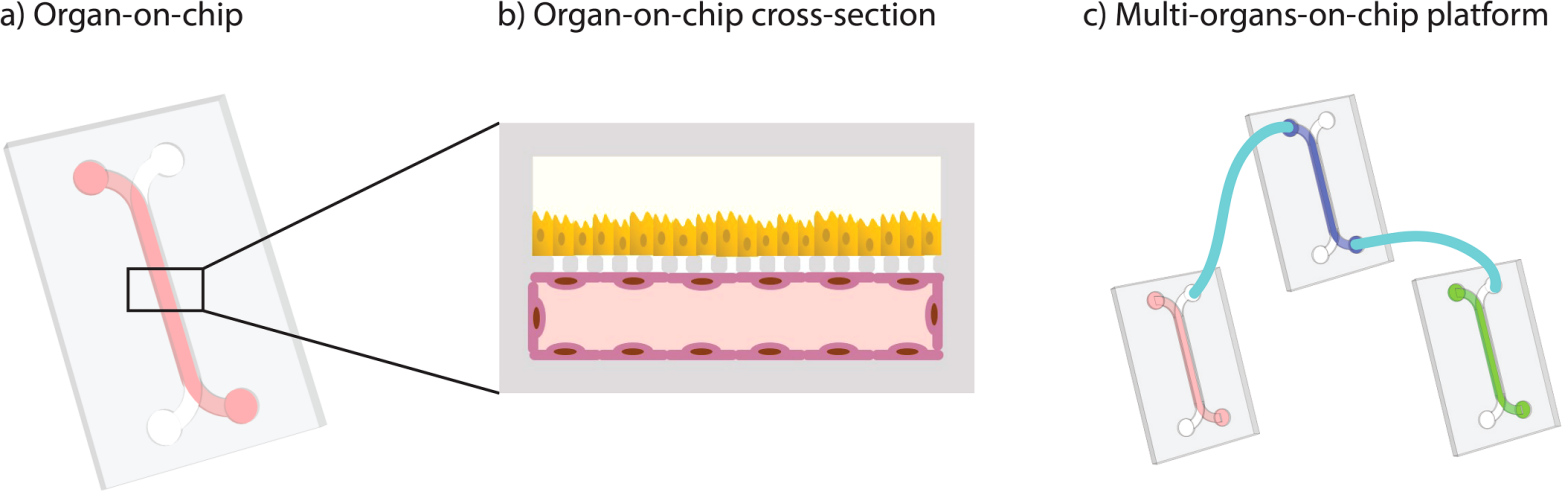
OoCs mimic functions of the human body in dynamic, microfluidic cell culture devices. (a) Schematic drawing of an OoC. (b) Cross section of a typical OoC reveals complex tissue co-cultures. (c) MOoC systems link multiple OoC devices to generate higher-level physiology and perform pharmacokinetics studies.

Organ-on-chips (OoC) are microfluidic cell culture devices that model aspects of organ-level functionality by mimicking microenvironmental aspects of tissues, including three-dimensional geometries and biophysical stimuli [Figs. 1(a) and 1(b)].^1,2^ In addition, sensors can be integrated for real-time monitoring of processes in these complex physiological models. For these reasons, OoCs have great potential for disease modeling, personalized medicine, and drug discovery,^3^ especially when paired with human stem cell technology.^4^ Furthermore, systems with multiple connected OoCs [Fig. 1(c)] are being developed to study organ–organ interactions.^5^ These multi-organs-on-chip (MOoC) systems are essential for drug development since drug and toxicity screening cannot realistically be confined to a single organ due to the drugs' metabolic pathways across several organs. At present, OoCs are used mostly by academic biomedical scientists to understand human-specific physiology, compound toxicity, and disease mechanisms.^6–11^ However, regulatory agencies, pharmaceutical companies, as well as food and cosmetic businesses show growing interest in using OoCs to test their products.^12–14^

As OoC systems are becoming increasingly complex, end-user adoption is often hampered by complicated device fabrication and operation of devices that are still at the proof-of-concept level. Without end-users, device development in academia often ends with the researcher's project and can lead to expensive re-inventing of the wheel by other developers in other labs. In contrast, commercially available devices are more user-friendly but often lack the physiological complexity of OoCs from academia. Moreover, end-users of modular OoC systems are limited to devices from one supplier or research group, since the components of these systems are not interchangeable due to the lack of interface standardization.

## II. TECHNOLOGICAL CHALLENGE

The OoC field can only reach its full potential if end-users can focus on using the OoC devices that best suit their research questions, without having to take into account which chips are available from a given supplier. There is widespread agreement in both academia and industry that OoC standardization is essential.^15–18^ The required standardization applies to many different aspects.^19^ Some aspects revolve around protocols, Good Manufacturing Practices (GMPs) of cells and devices, data standards, and compound sets for model validation, while others concern technological interfacing or form factors. Moreover, any form of standardization must also be compatible with the different funding schemes, protection of intellectual properties, and settings of research and development of both academia and industry.^20^ If successfully implemented, a widely accepted standardized system will greatly facilitate bringing new devices and functions from developers to end-users and shorten the path to commercialization. Innovation will thrive if developers can use standardized building blocks to make new setups.

In this Perspective review, we will focus primarily on the technical aspects related to the microfluidic operation of OoC systems. There are many stakeholders with varying interests even in this domain. Consequently, a key challenge of standardizing OoC devices lies in finely balancing the interests of all involved stakeholder groups. This means that OoC standardization cannot be mandated by a single party and must be carried by many participants to create benefits for all which are greater than the limitations inherently imposed by standardization. Here, we propose and discuss how an open platform strategy with standardized interface communication is a first step toward achieving this balancing act in an evolutionary, collaborative way.

## III. A PLATFORM FOR ORGANS-ON-CHIPS

### A. Defining an open organ-on-chip platform

**FIG. 2.**
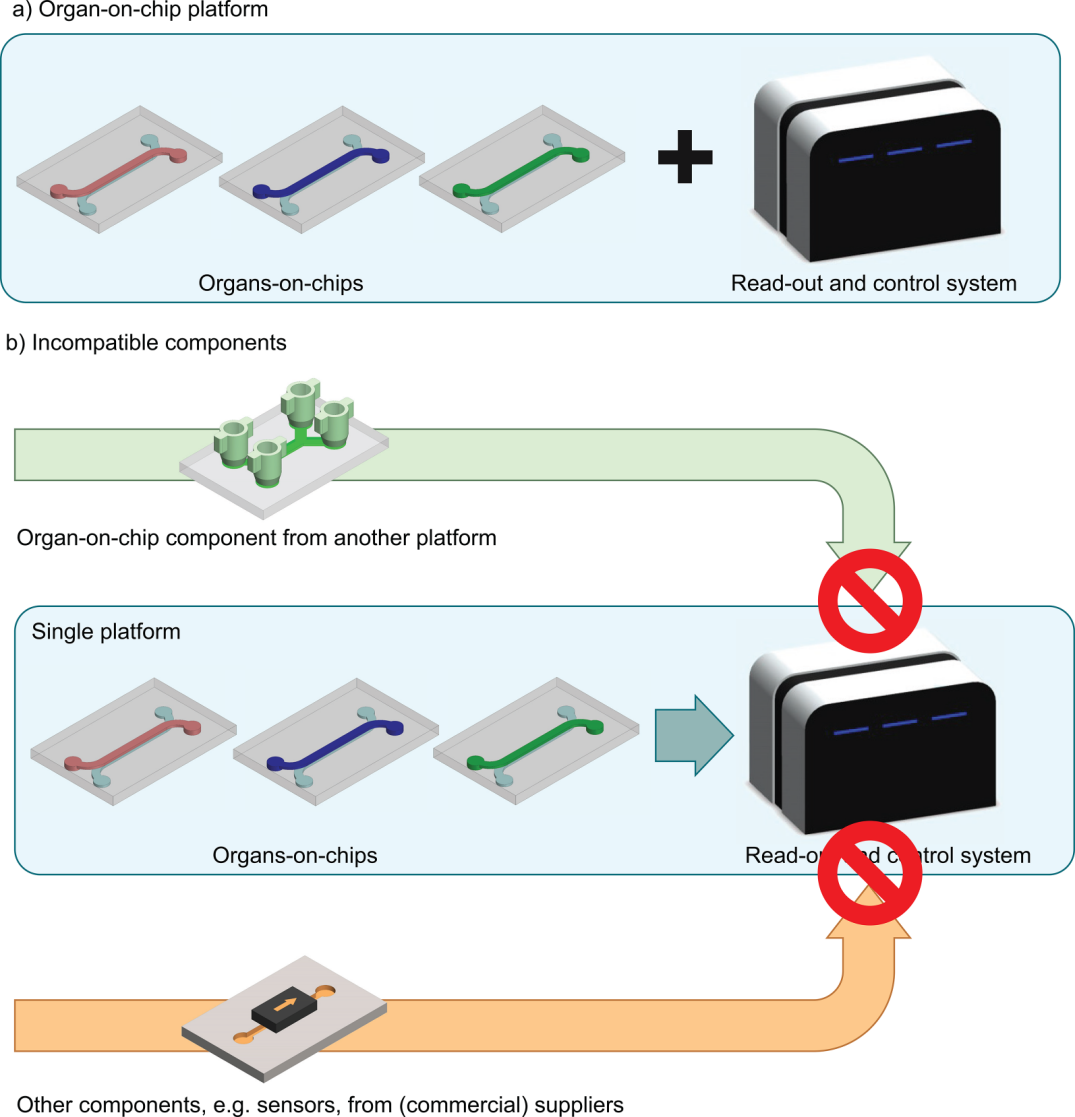
OoC technology platforms allow one to combine multiple components to achieve higher-level applications but their current impact is limited due to their closed nature. (a) Schematic representation of how OoC components can be combined into a higher-level system. (b) Schematic representation of how a closed platform has incompatible interfaces that prevent one to combine components of various platforms.

Platforms are used in many technologies (electronics, personal computing, social media, etc.). Although there is not one single definition, a *platform* generally comprises a set of components with shared interfacing rules from which new, higher-level products or applications can be created^21^ [Fig. 2(a)]. Many commercial and non-commercial platforms exist in the field of OoC.^17,18^ Some of these platforms focus on providing high flexibility through facilitating modular combinations of components,^22–24^ while others focus on a fixed set of components that integrate in a single way into a functional product. However, due to the incompatible interfacing rules of current OoC platforms, the end-user almost always stays confined to one platform's components for a certain application [Fig. 2(b)]. If components (e.g., a pump or a microfluidic chip) of one platform can be integrated into another platform, such components have a *cross-platform* design. Cross-platform applications would strongly facilitate the implementation and innovation in the OoC field because they allow developers and end-users to integrate existing solutions to more rapidly design OoC applications that are *fit-for-purpose*. In order to facilitate the development of cross-platform components, there is a need for *open* technology platforms in the OoC field.

**FIG. 3.**
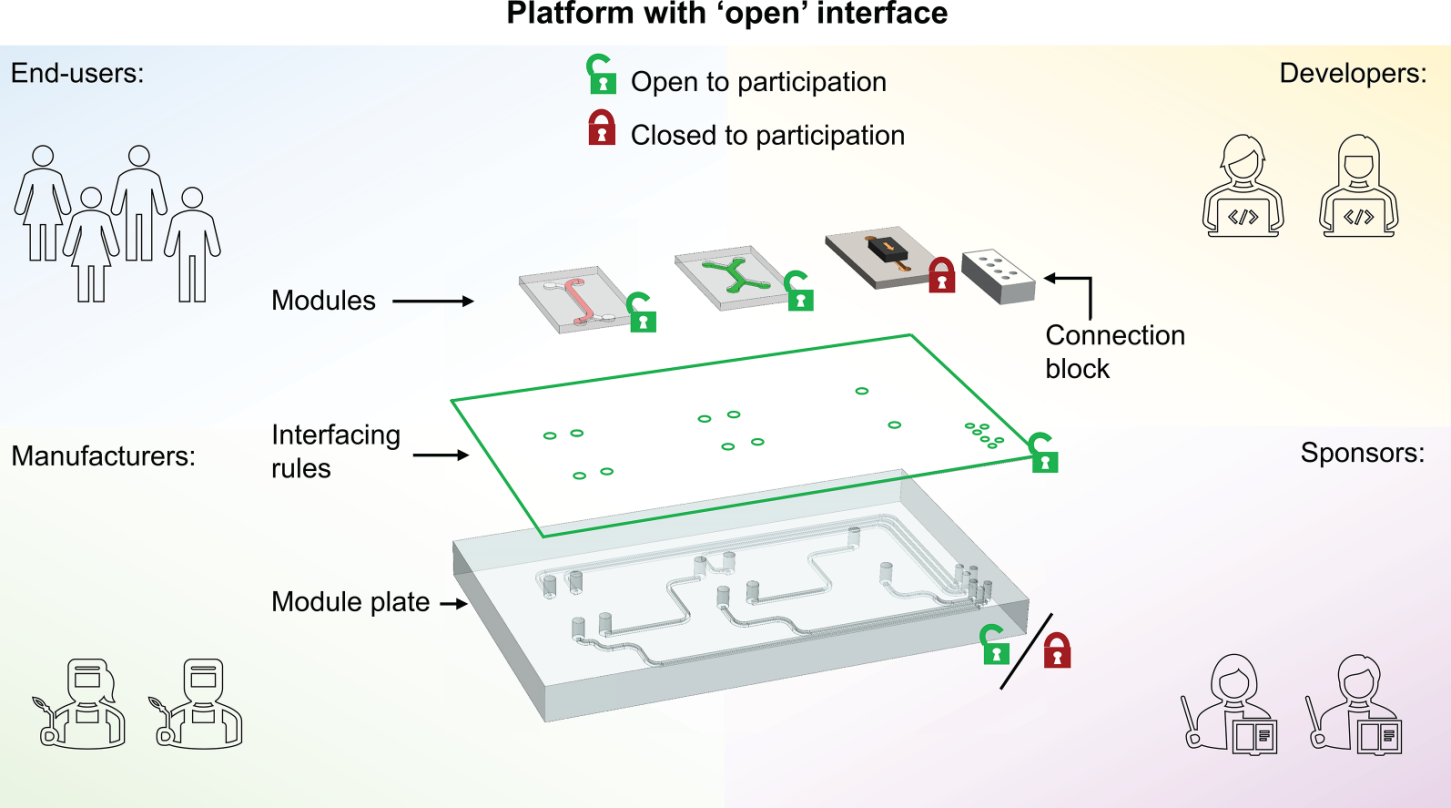
Schematic overview of an OoC platform, which has an open, standardized interface. Some components of the platform (e.g., some of the OoC modules or module plates) may be made closed to certain participant groups while the platform as a whole is still considered open.

Technology platforms can have both open and closed aspects. Eisenmann *et al*. define *open* as inviting contribution or encouraging participation with no or only few reasonable restrictions (e.g., accepting a license agreement).^25^ A platform can be either open or closed for anyone who wants to participate in the development and application of the platform. These participants can be divided roughly into four groups: end-users, developers, manufacturers, and sponsors (platform designers),^25^ as described in Table I and shown schematically in Fig. 3. For example, an OoC platform that is open for end-users can be used by anyone with minimal restrictions. In contrast, an OoC platform that is closed for this group can only be used by, for example, members of a certain institution or company. Another example is a platform that is open for developers, permitting anyone to develop components for the platform. In this case, the interfacing rules of the platform are openly available. In contrast, a platform can be open for end-users but closed for developers. In this scenario, anyone can use the platform but components can only be developed by groups of specific people. The case for manufacturers and sponsors is analogous. Since the role of sponsors is related to the core functionality of the platform (e.g., interface design), it is possible that any sponsor may introduce changes (open for sponsors) but these are then rejected by the overall community of participants. For example, if a sponsor of an OoC platform would develop an interface between components based on a specific type of tape or glue, this interface adjustment may be rejected because it is incompatible with other modules.

**TABLE I.** Participant groups in an open OoC platform.

Participant group	Role in an OoC platform	Examples
End-users	Perform experiments using the OoC platform	OoC researchers in academia and industry
Developers	Design and test new OoC components that are in keeping with the platform interface	OoC engineers in academia and industry. Engineers in industry might develop OoC devices, sensors or pumping systems which are proprietary, but can still be interfaced with other components of the platform.
Manufacturers	Fabricate any component of the OoC platform	Microfluidics companies
Sponsors	Document and update the platform interface	A consortium of stakeholders

If an OoC platform is fully open, it will facilitate collaboration between all four participant groups. It is important to note, however, that an open platform does not require all its individual components to be open to the same groups as well. Subsets, for example, OoC devices developed by a company, can be open for end-users but closed for developers and manufacturers. This way, companies can create proprietary components that are still compatible with an open platform. In fact, proprietary components may even be essential for long-term value creation and continued support of an open platform.

### B. Platform components and operation

In Sec. III B, the key components of a framework for user-friendly implementation of an open OoC platform are identified and described.

#### 1. Modules and interconnects

The goal of an open OoC platform is to enable developers and end-users to mix and match modules (e.g., OoC devices, micropumps, sensor chips, etc.) to be able to answer their research questions while leaving sufficient design freedom for module developers. Since the modules span a wide range of functions, from different OoCs to sensors and actuators, the interfaces need to be well-defined for all required domains of communication. These domains encompass most notably the routing of fluids between modules but also electrical and possible optical communication for, e.g., stimulation or sensor readout. However, some modules might be inherently incompatible in spite of their standardized interface (e.g., a peristaltic pump designed for high flow rates and an OoC containing cells that are highly sensitive to shear stress). Therefore, standardized documentation of the modules' properties and operation regimes (e.g., flow rate regimes, pressures, etc.) in a module database is essential.

#### 2. Module database

A comprehensive database containing all OoC, readout, and supporting modules is important for user-friendly and efficient experimental design as well as for subsequent system validation. Currently, potential OoC end-users can only search for OoC on separate companies' websites or indirectly by searching for the corresponding publications, which generally focus on obtained results rather than detailed device specifications. Finding and comparing devices in this way to make a well-informed choice of modules with which to build an overarching system would be very labor-intensive and time-consuming. In fact, the need for a microphysiological system database for model validation in drug testing has already been recognized by Gough *et al*.^26^ The authors created an online database currently containing 83 different devices ranging from conventional well plates to OoC, which have been used for compound screening on different organ models. The user can search the database using different criteria, such as organ type, compound, or disease model to obtain lists of corresponding devices with an image, short description, and link to the original publication. A similar database is essential for a platform in which several modules from different developers, both academic and industrial, are combined.

#### 3. Control and readout system

Current “proof of principle” OoC typically require lots of tubing, complex fabrication, and design-specific know-how. For end-users, this is a large obstacle that prevents them from focusing on using the OoC to answer their research questions. Therefore, an important part of establishing an open OoC platform is ensuring it is user-friendly. This is achieved by having both user-friendly physical components (“hardware”) and chip operating protocols (“software”). A good example is the so-called lab-on-a-disc.^27,28^ These microfluidic devices have a compact disc (CD)-format where the flow is driven by the centrifugal force during rotation of the device. Disc-specific protocols can be executed by adjusting the rotation speeds in a centrifuge. Device operation for the end-user means simply placing the disc in the “CD-player” and selecting the program supplied by the disc developer. Unfortunately, the lab-on-a-disc approach is poorly suited for a modular system since the rotation speeds of different functions have to be highly coordinated. Nonetheless, this level of user-friendliness is desirable. Therefore, an important step toward this goal is to identify essential equipment (e.g., for flow control and sensor readout) and provide a unified control and readout system with easy handling to reduce cumbersome connections and manual handling. The end-user can then connect this system to the OoC modules using a standardized fluidic connector, similar in principle to how devices in electronics can be connected by a USB cable.

#### 4. Platform software

Similar to the lab-on-a-disc system, the end-user should be able to select a desired program to run on the platform without having extensive knowledge of the operational aspects of every module. Therefore, the control and readout system needs to be able to automate protocols on the chip with parameter inputs (e.g., drug dosage) from the end-user. For simpler designs, basic functions (e.g., “start pump”) can be integrated into the software for the system. However, for complex modules, the automation scripts need to be supplied by the module developer. Consequently, a shared programming language has to be used, with a defined module application interface to ensure the operability of the final system.

### C. Toward an open platform: Translational organ-on-chip platform (TOP)

Recently, MFManufacturing—a consortium comprised of academic and industry partners from across Europe—took significant steps toward designing a standardized connection interface for microfluidics.^29–31^ The proposed platform architecture consists of a fluidic circuit board (FCB) acting as a baseplate and microfluidic building blocks (MFBBs) that are interfaced with the FCB in a standardized manner. In our view, the FCB is the microfluidic equivalent to the well-known electronic Printed Circuit Board (PCB) that serves as an internationally accepted standard in the realization of electronic circuits and which fast-tracked the industry's higher-level integration development to the complex systems of today. The MFBBs and FCB have standardized footprints, including footprints based on microscope slides and regular microtiter plates, and the fluidic inlets and outlets have a standardized pitch and diameter. These standards are based on a multi-stakeholder analysis and have been documented in an openly available ISO Workshop Agreement (IWA 23:2016).^32^ The MFBBs are interconnected through channels in the FCB to form a single, compact microfluidic system. In this way, a true “lab-on-a-chip” rather than a “chip-in-a-lab” can be created. Dekker *et al*. developed and characterized several MFBBs, forming an initial library.^31^ Later, Dekker *et al*. built a coulter counter that had an electric layer integrated into the FCB in addition to the fluidic layers. Using this layer, the FCB could be interfaced with a printed circuit board.^33^ Recently, we reported a platform following the same interfacing standards to parallelize three MFBBs designed for cell culturing,^34,35^ thereby demonstrating the potential of this platform for applications in the domain of OoC.

**FIG. 4.**
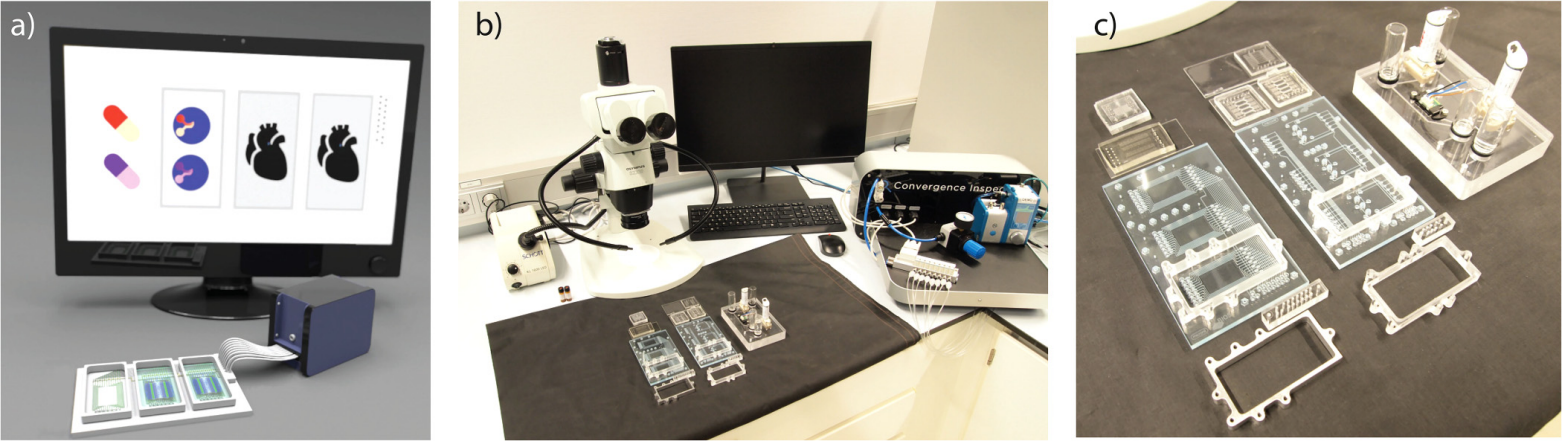
(a) Schematic overview of the components of the “Translational Organ-on-chip Platform” (TOP). Image adapted from the TOP animation video,^37^ which is licensed under the CC-BY license. (b) Example of a TOP setup. (c) Close-up of the collection of FCBs and MFBBs. From left to right, three FCBs in well plate format are shown. The left and middle FCB can be used to operate up to three complex MFBBs, which are shown beside the FCB. The right FCB is shown fully assembled with both in-house MFBBs and MFBBs based on commercially available products.

To date, the FCB and MFBB platform concept is being ported to the field of OoC with the support of the Human Organ and Disease Model Technologies (hDMT) consortium, which encompasses several Dutch universities, research institutes, and OoC start-up companies. This open platform is now known under the acronym “TOP,” which stands for Translational Organ-on-Chip Platform.^36^ MOoC MFBBs and various types of FCBs have been developed and are currently used for OoC research (Fig. 4). We have developed advanced FCBs based on polystyrene and thermoplastic elastomers to offer pneumatic control to connected chips but we have also developed more simple FCBs based on poly(methyl methacrylate) that offer re-circulation of the medium through an OoC module in between two fluid reservoir modules. The TOP platform has been conceptually expanded to include a readout and control system tailored to OoC research. Proof-of-concept experiments have demonstrated that TOP can be used to build a simple fluidic network to connect an in-house developed OoC to a commercially available flow sensor.

Importantly, TOP is supported by a network of end-users, developers, and manufacturers from both academia and industry, which is essential for a broad adoption of the platform and its interfacing standards. Currently, several research projects using TOP are in progress including the development of new FCBs as well as gut-on-a-chip, heart-on-a-chip, and cancer-on-a-chip MFBBs. TOP is only offering the first steps of an open OoC platform since all development so far has focused almost exclusively on modules and interconnects. However, the development of control systems and software (as outlined in Sec. III B) will play an important role in the future.

## IV. SUMMARY AND OUTLOOK

**FIG. 5.**
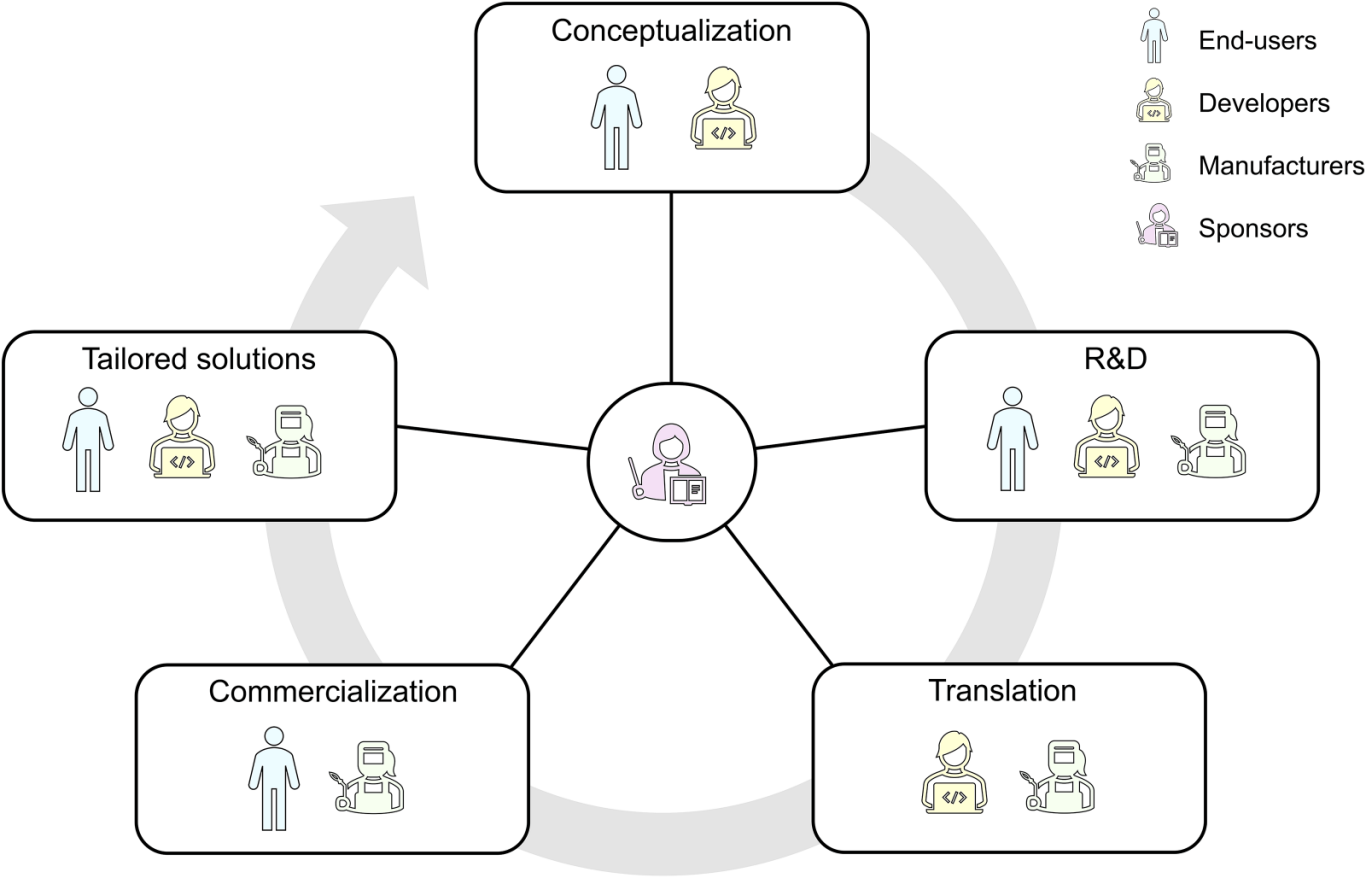
Life cycle of a typical project, showing the involvement of the four different participant groups at each stage.

An open OoC platform can be seen as the physical manifestation of networking between different OoC researchers. The platform-mediated network facilitates bringing new devices from the developer's lab into the “real world” application of the end-user, allowing both parties to fully use their respective expertise while still being part of a multidisciplinary project. For the OoC field, a standardized and automated way of operating devices and an accompanying database will also facilitate implementing methods to have models quickly tested by end-users and academics without needing to set up device-specific control and readout systems. If we want to facilitate standardization in OoC by the development of an open platform, it will be essential that the platform finds widespread support. An ongoing dialogue between developers, manufacturers, users, and sponsors will be important to promote the use of the platform (Fig. 5). However, perhaps even more importantly, a platform needs to demonstrate its added value by enabling new applications. We know from other sectors, e.g., the field of microelectronics, how immensely powerful standardization can be in accelerating the development and application of technology. In the coming years, the field of OoC will need to come together to accomplish a similar revolution. It will require technical innovation, as well as significant investments from all stakeholders, but it is clear that the future of the field depends on it.

## Data Availability

Data sharing is not applicable to this article as no new data were created or analyzed in this study.
